# Revealing the Underlying Mechanism of *Acacia Nilotica* against Asthma from a Systematic Perspective: A Network Pharmacology and Molecular Docking Study

**DOI:** 10.3390/life13020411

**Published:** 2023-02-01

**Authors:** Taghreed S. Alnusaire, Sumera Qasim, Mohammad M. Al-Sanea, Omnia Hendawy, Ambreen Malik Uttra, Shaimaa R. Ahmed

**Affiliations:** 1 Biology Department, College of Science, Jouf University, Sakaka 72341, Saudi Arabia; 2 Department of Pharmacology, College of Pharmacy, Jouf University, Sakaka 72341, Saudi Arabia; 3 Pharmaceutical Chemistry Department, College of Pharmacy, Jouf University, Sakaka 72341, Saudi Arabia; 4 Department of Clinical Pharmacology, Faculty of Medicine, Beni-Suef University, Beni-Suef 11562, Egypt; 5 Department of Pharmacology, College of Pharmacy, University of Sargodha, Sargodha 40100, Pakistan; 6 Department of Pharmacognosy, College of Pharmacy, Jouf University, Sakaka 72341, Saudi Arabia; 7 Department of Pharmacognosy, Faculty of Pharmacy, Cairo University, Kasr El-Aini Street, Cairo 11562, Egypt

**Keywords:** *Acacia Nilotica*, asthma, network pharmacology, molecular docking

## Abstract

*Acacia Nilotica* (AN) has long been used as a folk cure for asthma, but little is known about how AN could possibly modulate this disease. Thus, an in-silico molecular mechanism for AN’s anti-asthmatic action was elucidated utilizing network pharmacology and molecular docking techniques. DPED, PubChem, Binding DB, DisGeNET, DAVID, and STRING were a few databases used to collect network data. MOE 2015.10 software was used for molecular docking. Out of 51 searched compounds of AN, eighteen compounds interacted with human target genes, a total of 189 compounds-related genes, and 2096 asthma-related genes were found in public databases, with 80 overlapping genes between them. AKT1, EGFR, VEGFA, and HSP90AB were the hub genes, whereas quercetin and apigenin were the most active components. p13AKT and MAPK signaling pathways were found to be the primary target of AN. Outcomes of network pharmacology and molecular docking predicted that AN might exert its anti-asthmatic effect probably by altering the p13AKT and MAPK signaling pathway.

## 1. Introduction

Asthma has been described as a common long-term inflammatory condition of the respiratory airways. Airway inflammation, hyperresponsiveness, and remodeling are the prime manifestations of asthma. The recurrence of the disease, along with the high expense of available treatment, make it life-threatening sometimes [1]. Many treatment options are available for managing asthma, but all are preventive therapies with associated side effects. Inhaled corticosteroids (ICS) or ICS coupled with long-acting β2-adrenergic agonists are primarily employed for managing asthma. However, in some circumstances, corticosteroid overload poses a high risk of glucocorticoid-related adverse effects and makes it extremely difficult to manage the onset of asthma. Thereby it is a need of time to establish new treatment strategies for managing asthma [2]. Since ancient times, traditional herbal medicinal agents have been utilized to treat various ailments, but their use has significantly increased in the last few years. Eighty percent of the world’s population relies on phytotherapeutic agents to meet their primary healthcare needs, and 11 percent of important medications are plant-based [3]. In middle income countries especially Asia and Africa, herbal medicines are used by 80 percent of the people [4].

*Acacia Nilotica* (AN) possesses a wide range of biological attributes. *Acacia* trees are abundant in Saudi Arabia, India, Sudan, Pakistan, Egypt, and Sri Lanka and are used by ordinary people as a nutritional supplement and medicinal agent [4]. Adding this antioxidant-rich medicinal herb to the diet can help prevent oxidative stress, a factor involved in the pathophysiology of several disorders. *Acacia Nilotica* possesses anticancer, antispasmodic, antipyretic, anti-diabetic, antifungal, antiviral, antibacterial, and antihypertensive properties [5]. In Pakistan, AN has traditionally been used for asthma management [6]. The lack of pharmacological records on the anti-asthmatic activity of AN necessitated this investigation, which used network pharmacology and molecular docking techniques to elucidate its anti-asthmatic potential.

**Figure 1:**
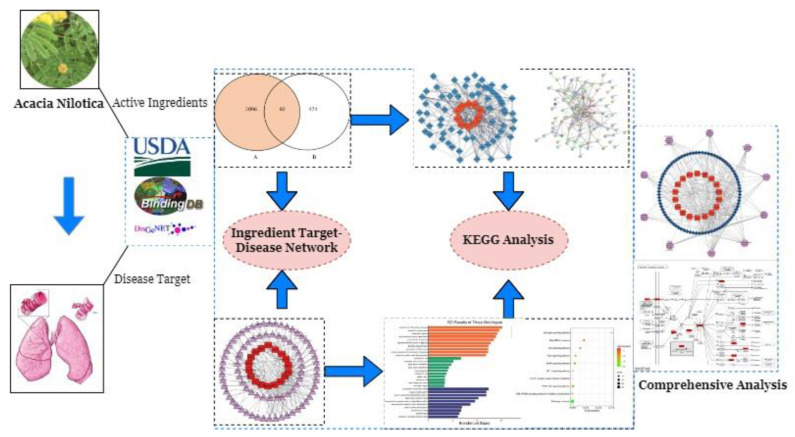
The overworkflow for elucidation of anti-asthmatic potential of *Acacia Nilotica*.

The network pharmacology approach allows for studying the interaction among compounds, genes, and diseases. This provides a better understanding of the contribution of any particular test compound in the treatment or prevention of any specific disease by targeting the genes involved in disease pathogenesis. Network pharmacology employs a multifunctional approach to elucidate the mechanism of action of multiple compounds rather than concentrating on interactions between a single molecule and a single target. This makes it possible to discover novel, natural pharmacological treatments and preventative measures with novel mechanisms of action [7]. Thereby, in the current investigation network pharmacology approach investigated the likely mechanism of AN in the treatment of asthma. The overall workflow for the entire study is shown in Figure 1.

## 2. Results

### 2.1. Active Compound’s Screening of AN

The literature retrieval and DPED database found 51 compounds in AN, the names and molecular formulae reported in Supplementary Table S1. ADMET (Absorption, distribution, metabolism, excretion, and toxicity) screen of 51 compounds revealed that 18 compounds were “Accepted”, suggesting they had good potential as active components. The effectiveness of selected compounds as leads was confirmed by conducting an ADMET analysis. A freely available web server, pkCSM, was used to predict the ADMET characteristics of selected compounds (Table 1). In the ADMET assessment, anticipated values for absorption, such as water solubility (log mol/L), intestine solubility (% absorbed), and skin permeability (log Kp), supported the significant therapeutic potential of particular drugs. According to research, substances with good absorption characteristics can passively penetrate the intestinal wall and reach the desired target. Compared to standard values (absorption > 30%, skin permeability > −2.5 log Kp), all the compounds displayed appropriate intestinal solubility and skin permeability. Additionally, all screened compounds’ Blood Brain Barrier (BBB) and Central Nervous System (CNS) permeability values were equivalent to the standard values (i.e., >0.3 to −1 log BB and >−2 to −3 logPS, respectively). Moreover, CYP3A4 and CYP1A2, isoforms of cytochrome P450, validated their computational metabolic function with inhibitory potential. Based on total clearance (log mL/min/kg), Ames toxicity, maximum tolerated dose (MTD), and LD50 values, the excretion and toxicity predicted values similarly supported the drug-like behavior of these substances. The Ames toxicity prediction further validated selected compounds’ non-mutagenic and nontoxic behavior. Negative behavior related to hepatotoxicity and skin sensitivity also demonstrated these effects’ non-toxic and less sensitive nature. These hypothetical ADMET findings indicated that the compounds had a good lead-like potential for further research.

### 2.2. Potential Targets Identification

**Figure 2:**
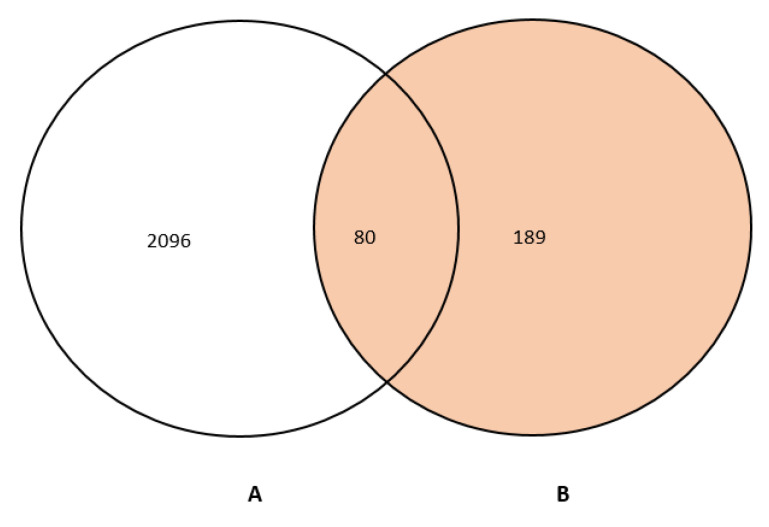
A Venn diagram showing overlapping genes between 2096 asthma-related genes (**A**) and 189 compounds related genes (**B**).

Through the STITCH and BindingDB databases, 189 genes were targeted by 18 phytoconstituents of AN (Supplementary Data Table S2). 2096 Asthma-related genes were retrieved from the DisGeNet database (Supplementary Data Table S3). Mapping 189 active ingredient targets with 2096 asthma targets yielded 80 similar targets (Figure 2, Supplementary Table S4), regarded as potential *Acacia Nilotica* asthma targets.

### 2.3. Phytoconstituents-Target Network

**Figure 3:**
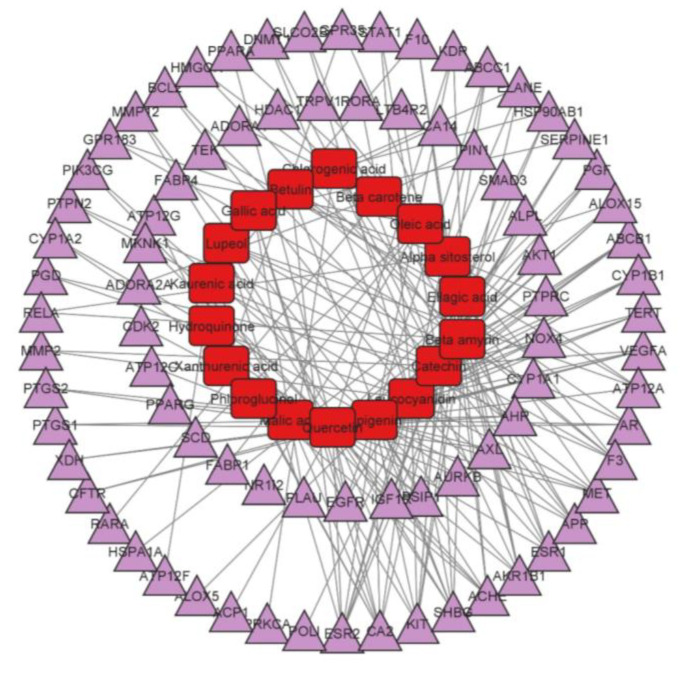
Network with 99 nodes and 206 edges linking 18 compounds of *Acacia Nilotica* with 80 genes of Asthma. The red-colored nodes in the network’s center indicate AN components, while the purple-colored nodes reflect asthma’s potential targets.

To investigate the relationship between active compounds and prospective targets, Cytoscape was used to build a network between potential targets and active compounds. A compound-target network was constructed with 80 possible target genes and 18 active compounds in AN. Using a “network analyzer”, we discovered that the compound-target network has 206 edges and 99 nodes (Figure 3).

In a compound-target network, the degree of 18 active compounds was examined (Table 2). Flavones and terpenoids have the highest degree compared to other classes of compounds. The contribution difference of each compound and gene to the anti-asthmatic action of AN may be determined using the degree values of each compound and gene (Table 2). The highest degree-bearing active ingredients of AN against asthma were apigenin and quercetin, which were linked to 35 genes. A network analysis of target-compound interactions reveals that one active ingredient can affect multiple targets while the same target can interact with various active compounds. This reflects the AN’s multi-target and multi-component efficacy against asthma.

### 2.4. PPI Network Analysis

**Figure 4:**
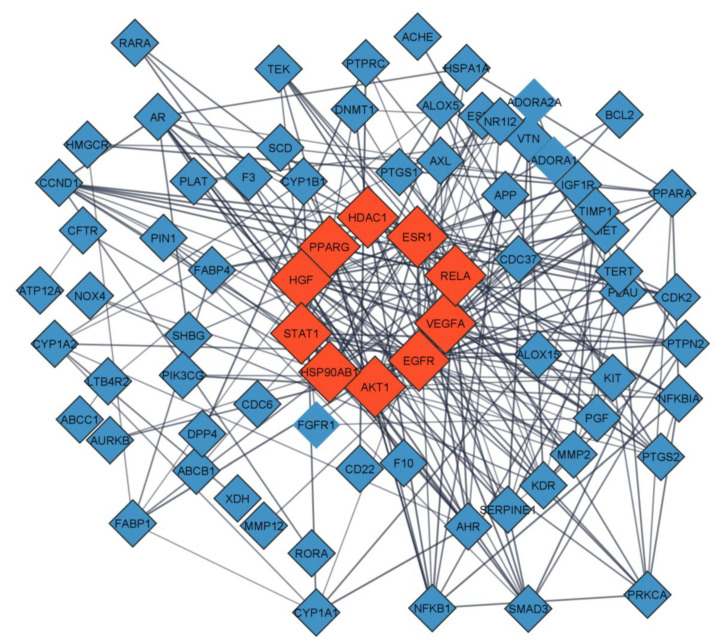
Protein-protein interaction (PPI) network analysis.

The association between the overlapping genes are revealed through protein-protein interaction studies. Interrelationship between genes involved in a particular disease pathophysiology can be established by PPI network. These overlapping genes were submitted to STRING version 11.5 and high confidence protein interaction data with a score >0.7 was chosen for PPI network building (Figure 4). The highest degree value between genes could be considered as their strong correlation and hence their importance in disease development and progression.

Cytoscape was used to visualize the PPI network, and 76 nodes and 227 edges were found (Figure 4). The Hub genes were discovered using the CytoHubba plugin. There are twelve topological ways of analysis in the CytoHubba. From these 12 techniques, the degree technique was employed to predict Hub genes. The highest degree suggests that the targets are more interconnected, suggesting that it may be a target responsible for the biological impact of the compound. AKT1 (23), EGFR (21), VEGFA (18), RELA (16), ESR1 (16), HDAC1 (14), STAT1 (13), PPARG (12), AR (13), and HSP90AB1 (11) are among the top 10 hub genes with high degree values in the network (Figure 4).

### 2.5. GO Analysis and KEGG Pathway

**Figure 5:**
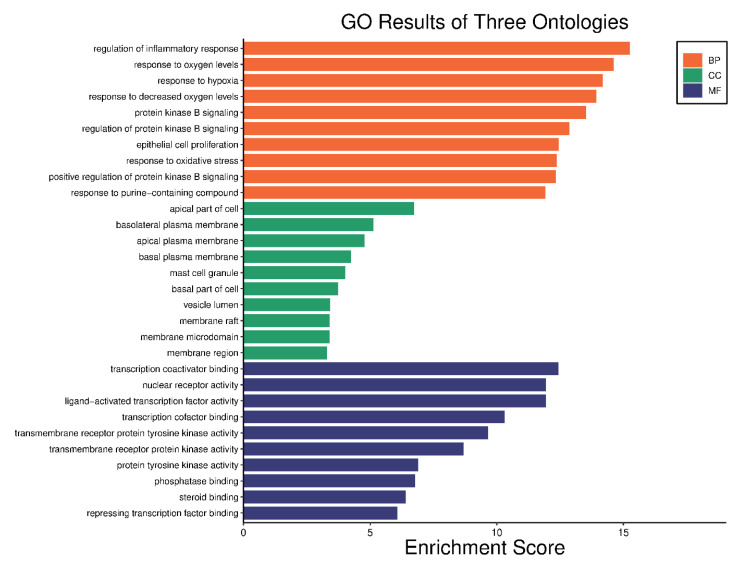
Gene Ontology (GO) enrichment analysis of the target proteins. Biological process (orange), molecular function (blue) and cellular component (green).

GO annotations and KEGG pathway analysis were performed on 80 anti-asthmatic targets to demonstrate the molecular mechanism of AN in the treatment of asthma. Go analysis of target genes of AN’s targets was related to the regulation of inflammation, response to oxygen levels, response to hypoxia, protein kinase B signaling, and many others. The cellular compartment (CC) includes the apical part of the cell, basolateral plasma membrane, and mast cell granules. At the same time, molecular function (MF) provides transcription co-activity binding, nuclear receptor activity, ligand-activated transcription factor activity, and many others, as shown in Figure 5.

**Figure 6:**
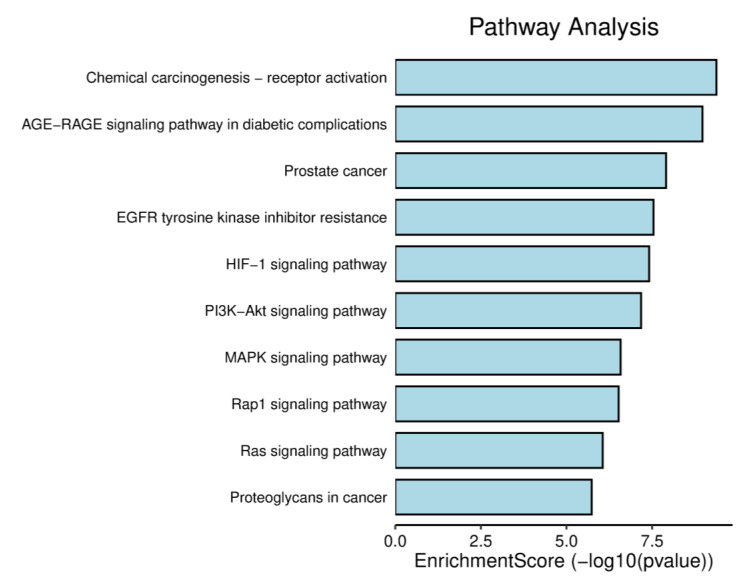
Bar chart of top 10 signaling pathways.

**Figure 7:**
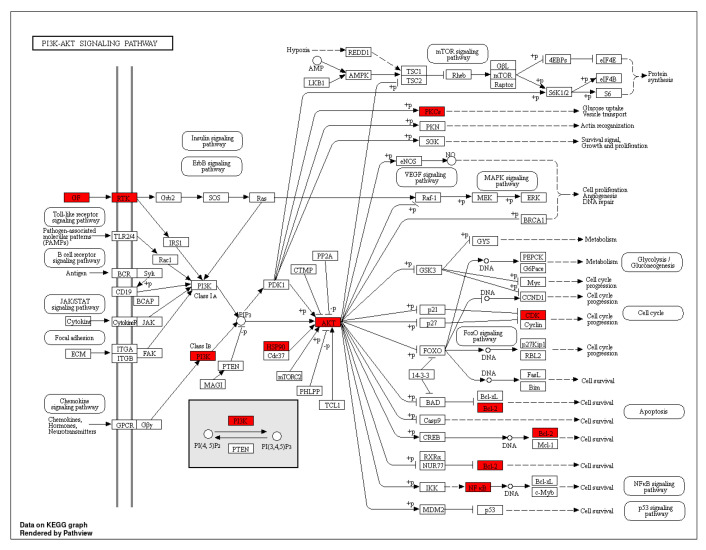
p13-AKT signaling pathways. Red boxes represent the genes of the pathway targeted by compounds of AN.

The top 10 GO annotations (BP, CC, and MF) (Figure 5) and ten highly enriched KEGG pathways (Figure 6) were chosen using the cutoff value of *p* ˂ 0.05. According to KEGG analysis, as shown in Figure 6, most of the target genes were involved in pathways chemical carcinogenesis-receptor activation, AGE-RAGE signaling pathway, EGFR tyrosine kinase inhibitor resistance, P13K-Akt signaling pathway, and so forth. A literature review of these pathways shows that the P13K-Akt signaling pathway (Figure 7) has a significant contributory role in asthma pathophysiology; thereby, targeting this pathway by AN’s compounds might be the possible mechanism of its anti-asthmatic action.

### 2.6. Analysis of Target-Pathway-Compound Network

**Figure 8:**
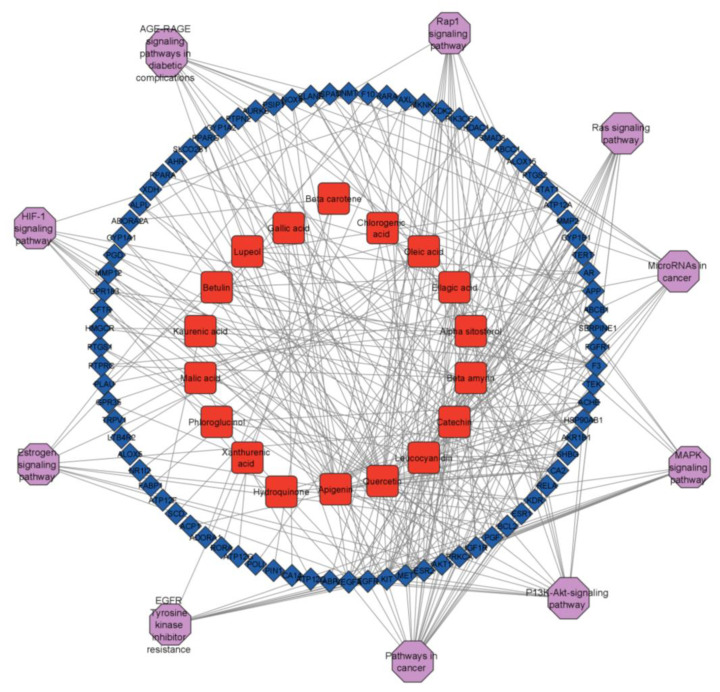
Network of compound-target-pathway where blue colored rhomboid boxes represents targets, red square nodes represent compounds and purple colored octagons represents pathways.

Network analysis was used to investigate the mechanism of AN in asthma. DAVID analysis was used to determine the top 10 enriched pathways, and Cytoscape was used to generate the target-pathway-compound network. The network contained 110 nodes and 335 edges, with 18 active components, 80 potential targets, and ten critical pathways (Figure 8. The targets of AN active components show coordination with many paths and are interconnected, characterized by multi-target, multi-component, and multi-pathway physiognomies. AKT1 was found to be implicated in several pathways among all of the genes studied.

### 2.7. Molecular Docking

**Figure 9:**
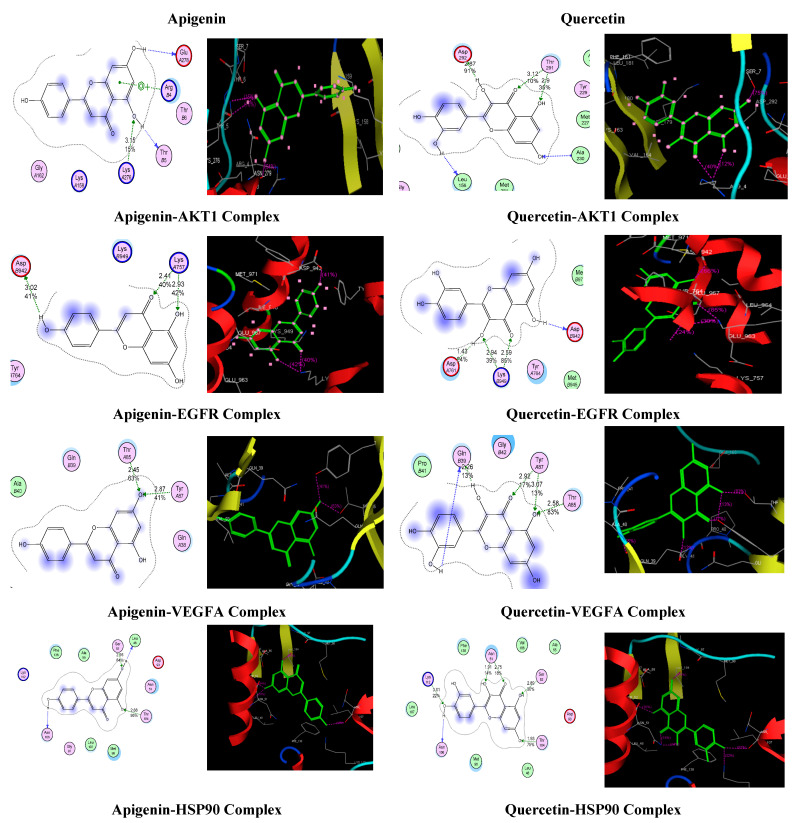
Apigenin and Quercetin possible binding modalities in the binding pockets of the specified targets. A green dotted line shows side-chain proton acceptor/donors, while a purple dotted line shows metal or ion contact. The blue and red circles, which represent basic and acidic amino acids, respectively. Because they have been exposed to solvents, some amino acids have a blue background. Additionally, the blue coloring of the ligand atoms in front of them indicates solvent exposure.

For the molecular docking, the four target genes, AKT1, EGFR, VEGFA, and HSP90AB, were selected based on KEGG analysis results in the asthma pathway. The topmost active compounds, apigenin and quercetin were docked with these chosen targets. The two crucial factors were used while analyzing docking results: (i) the best-docked pose binding energy prediction using MOE (Molecular operating environment) scoring system and (ii) Hydrogen bond information of the top-ranked pose. Each compound was docked in 10 distinct positions throughout the docking run. The recovered molecules were first sorted using the pre-validated methodology, and then the visualization approach was used to determine the inhibitor binding mode that is best based on the inhibitor’s critical interactions with the active site residues. Table 3 summarizes the docking information for the top-ranked poses. Docking score and binding mode pattern of both compounds showed significant interaction with the mentioned targets. Docking score of less than −5 kj/mol confirms the strong interaction of both compounds with selected targets. Figure 9 shows the docking results of four targets with apigenin and quercetin.

## 3. Discussion

Chronic inflammation is a hallmark of asthma, and airway immune inflammation contributes significantly to asthmatic symptoms such as inflammation, hyperresponsiveness, reversible airway restriction, and remodeling [8]. Traditional medicines have gained significant attention due to their better efficacy and fewer side effects [9]. GO, pathway enrichment analysis, and molecular docking was used in this study to demonstrate *Acacia Nilotica*’s mechanism in treating asthma.

Eighteen compounds were associated with AN’s ability to alleviate asthma symptoms, including four flavonoids, three terpenoids, three phenolic acids, two phenolics, one diterpene, one sterol, and carotenoid. AN’s therapeutic impact on asthma appears primarily due to terpenoids and flavonoids. Compounds-genes network analysis indicated that quercetin and apigenin were the most potent active components against asthma. A literature review of the anti-asthmatic activity of quercetin and apigenin further confirmed this finding. Both compounds displayed significant anti-asthmatic activity in animal models of asthma [10,11,12,13]. Above mentioned studies provided evidence of the anti-asthmatic potential of apigenin and quercetin. Still, they needed to include detailed mechanistic studies about their action on particular pathways involved in the pathophysiology of asthma. Thus current work gave a probable mechanism of action of these compounds that contribute to their anti-asthmatic potential. Results from PPI network analysis using Cytoscape showed the top 10 hub genes to be AKT1, EGFR, VEGFA, RELA, ESR1, HDAC1 (14), STAT1 (13), PPARG (12), AR, and HSP90AB1 (11). Based on KEGG pathway analysis along with PPI network analysis, it was concluded that four genes targeted by AN active compounds, including AKT1, EGFR, VEGFA, and HSP90AB, have the most prominent role in the anti-asthmatic potential of AN. Asthmatic airway remodeling is characterized by the activation of airway smooth muscle by Akt [14,15]. One of the studies found that inhibiting AKT activation reduced airway hyperactivity, airway inflammation, and airway remodeling in an animal model of asthma [16]. Airway remodeling, airway hyper mucus secretion, and immunological responses to airway inflammation are all affected by EGFR and VEGFA [17,18,19,20]. HSP90AB1 is linked to the immunological response, where it can activate macrophages to generate inflammatory markers, including IL-6 and TNF-α [21]. Thus these four target genes contribute to asthma onset and progression. Quercetin and apigenin demonstrated excellent binding capabilities with hub targets such as AKT1, EGFR, VEGFA, and HSP90AB1 in molecular docking studies. Both compounds bind in the active binding site of target proteins in the same way as the co-crystallized ligand binds. 

Seventy-eight signaling pathways, targeted by 18 compounds of AN and 80 target genes, including the PI3K-AKT pathway, MAPK signaling pathway, and Ras signaling pathway, were revealed by KEGG analysis. PI3K-AKT signaling pathway was found to be the highly enriched pathway targeting 16 overlapping genes compared to the other screened pathways. AKT pathway has been shown to reduce cell proliferation in airway smooth muscle cells in asthmatic conditions [1]. PI3K signaling molecule is involved in practically every facet of asthma pathogenesis. Inhibition of PI3K reduces mucus production, mast cell degranulation, and recruitment of immune cells and promotes bronchodilation. These attributes have therapeutic efficacy in managing asthma; therefore, AN might exert its anti-asthmatic action by down-regulating the P13K-AKT signaling pathway. The second most prominent signaling pathway with a central role in the pathobiology of asthma includes the MAPK signaling pathway targeting 14 asthmatic genes. By modulating the expression of pro-inflammatory genes such as TNF-α and IL-6, MAPK signaling pathways help modulate asthmatic lung inflammation and immunological responses. Proinflammatory cytokines such as interleukin-1β, interleukin-6, and tumor necrosis factor (TNF)-α may activate the MAPK signaling pathway leading to increased inflammation [22]. Thus, based on network pharmacology and molecular docking analysis, *Acacia Nilotica*’s anti-asthmatic potential is probably due to its immunomodulatory and anti-inflammatory potential. In this study, the molecular mechanism of *Acacia Nilotica* was clarified using network pharmacology and molecular docking techniques. The present study had some limitations. The current work is based on an in silico approach; detailed in vivo and in vitro studies are required to validate these findings and point out the precise mechanism of action.

## 4. Materials and Methods

### 4.1. Database Construction and ADMET Analysis of AN Phytoconstituents

The bioactive ingredients of AN were searched using Dr. Duke’s Phytochemical and Ethno botanical Database (DPED), Web of Science, and Google Scholar, while the SMILES and molecular formulas of constituents were discovered using PubChem (https://pubchem.ncbi.nlm.nih.gov/) accessed on 23 March 2022 [23]. pkCSM (https://biosig.lab.uq.edu.au/pkcsm/prediction) was used to conduct ADMET analysis on selected constituents, accessed on 27 March 2022. Active components were selected when the ADMET evaluation results were deemed acceptable. Compounds which have appropriate intestinal absorption, excretion and no toxicity were selected for further evaluation.

### 4.2. Target Genes Associated with Asthma and Selected Compounds

Binding DB (https://www.bindingdb.org/bind/index.jsp), accessed on 28 March 2022, was used using the “homo sapiens” setting to predict target genes for selected compounds based on SMILES. The “minimum needed interaction score” was set to “high confidence (0.700)” during Binding DB prediction. The public database DisGeNET (http://www.disgenet.org/, accessed on 1 April 2022) was used to identify asthma-related target genes. A Venn diagram was used to identify and illustrate the overlapping genes between the compounds and the asthma target genes [24].

### 4.3. Interactions between Compounds and Overlapping Genes: Network Construction

Cytoscape ver. 3.9.1 (https://cytoscape.org/, accessed on 29 November 2022) was used to construct, display, and analyze the network of interactions based on the Binding DB prediction results for constituents and overlapping genes [24]. Nodes in the network indicate bioactive components and genes, while edges show interactions between compounds and genes. Anti-asthmatic AN components and hub genes were identified by analyzing the network’s topological structure and setting the “Degree value” of compounds or genes, respectively [25]. A compound’s or a gene’s degree value represents how many phytoconstituents or genes are present in a network. AN’s therapeutic effect on asthma is enhanced if a compound targets more disease-inducing genes.

### 4.4. Building a Protein-Protein Interaction Network

An online database called STRING, version 11.5, (https://string-db.org/, accessed on 29 November 2022) was used to gather information on protein-protein interactions between the target proteins of selected AN components (PPI). The website calculated a score for each protein’s mutual information. The stronger the contact between the two target proteins, the higher the score. Since high confidence data >0.7 were used to ensure accuracy and reliability, the study was considered reliable. The obtained protein interaction data were imported into the Cytoscape 3.9.1 application to generate a PPI protein interaction network. The CytoHubba plug-in was employed for the identification of Hub genes [26]

### 4.5. Target Protein Gene Ontology (GO) and KEGG Enrichment Analysis

The selected target genes were analyzed for GO and KEGG analysis by using (David) v 6.8 software. Cellular components (CC), molecular functions, biological processes were analyzed for GO analysis. KEGG pathway enrichment analysis was done to predict a possible molecular mechanisms of AN against asthma. GO KEGG pathway bar charts were made with SRPLOT (http://bioinformatics.com.cn/, accessed on 29 November 2022) [27].

### 4.6. Target-Pathway-Compound Network Construction

It is possible to perform a pathway enrichment analysis using KEGG as well as pathway functional annotations for a given gene set. The Cytoscape 3.9.1 software was used to create the compound–target–pathway network seen in Figure 8 based on results from the DAVID database. The development of a network showed the features of numerous AN components, targets, and pathways.

### 4.7. Molecular Docking

MOE 2015 (Molecular operating environment) was employed as the molecular docking tool. Crystal structure of AKT1, EGFR, VEGFA and Hsp90AB1 were found in the protein data bank (PDB ID: 6HHG, 6Z4B, 7VSW and 3NMQ). The builder tool of MOE was used to prepare the structures of apigenin and quercetin. The MOE tool’s Energy minimization algorithm was used to reduce the energy of the protein molecule. The following variables were used to minimize energy; 0.05 Gradient, MMFF94X + Solvation Force Field, and Current Geometry Chiral Constraint. When the root mean square gradient dropped below 0.05, energy minimization was stopped. Ten distinct docked conformations for each compound were produced once the active site was chosen. For binding pattern analysis, the compound’s lowest energy conformation was chosen. The minimized protein structure was used as the docking template [27].

## 5. Conclusions

For the first time, the active components and mechanisms of AN’s anti-asthma properties were studied using network pharmacology. According to network pharmacology results, quercetin and apigenin were found to be the most enriched constituents of AN, and the AKT1, EGFR, VEGFA, and HSP90AB were enriched hub genes, respectively. The fundamental mechanism of AN against asthma was the deactivation of the p13AKT signaling pathway, which may aid in the prevention and progression of asthma and other inflammatory diseases. Based on network pharmacology and molecular docking-based prediction, it can be concluded that AN might be considered a natural source of asthma management; however, detailed mechanistic studies are required to validate this hypothesis. 

## Figures and Tables

**Table 1:** ADMET Analysis of selected compounds of *Acacia Nilotica*.

Compound	Pubchem ID	Absorption			Distribution		Metabolism		Excretion	Toxicity				
		WS (log mol/L)	IS (% abs)	SP (log Kp)	BBB (log BB)	CNSP (log PS)	CYP3A4 Inhibitor	CYP2C19 Inhibitor	TC (log mL/min/kg)	Max Tolerated Dose	ORAT (LD_50_)	HT	SS	AMES
Catechin	9064	−3.11	68.82	−2.37	−1.054	−3.298	No	No	0.183	0.438	2.428	No	No	No
Leucocyanidin	71629	−2.98	56.71	−2.37	−0.91	−3.213	No	No	−0.072	0.446	2.394	No	No	No
Alpha sitosterol	9548595	−6.66	94.87	−2.61	0.782	−1.554	Yes	No	0.585	−0.578	2.56	No	No	No
Apigenin	5280443	−3.32	93.25	−2.37	−1.734	−2.061	No	Yes	0.566	0.328	2.45	No	No	No
Beta amyrin	73145	−6.531	93.73	−2.11	0.667	−1.773	Yes	No	−0.044	−0.56	2.478	No	No	No
Beta carotene	5280489	−7.39	91.73	−2.21	0.938	−1.094	Yes	No	1.061	−0.379	2.073	No	No	No
Betulin	72326	−5.446	94.53	−2.32	−1.295	−2.035	Yes	No	0.236	−0.794	2.699	Yes	No	No
Chlorogenic acid	1794427	−2.449	36.37	−2.35	−1.407	−3.856	No	No	0.307	−0.134	1.973	No	No	No
Ellagic acid	5281855	−3.181	86.68	−2.35	−1.272	−3.533	No	No	0.537	0.476	2.399	No	No	No
Gallic acid	370	−2.56	43.37	−2.35	−1.102	−3.74	No	No	0.518	0.7	2.218	No	No	No
Hydroquinone	785	−0.762	86.85	−2.18	−0.318	−2.076	No	No	0.52	0.707	2.008	No	Yes	No
Kaurenic acid	73062	−3.096	100	−2.35	0.05	−1.602	Yes	No	0.506	0.046	2.031	Yes	No	No
Lupeol	259846	−5.861	95.78	−2.44	0.726	−1.714	Yes	No	0.153	−0.502	2.563	No	No	No
Malic acid	222656	−1.381	13.83	−2.35	−1.788	−3.523	No	No	0.81	1.212	1.818	No	No	No
Oleic acid	445639	−5.924	91.82	−2.25	−1.168	−1.654	Yes	No	1.884	−0.81	1.417	No	Yes	No
Phloroglucinol	359	−1.408	83.54	−2.51	−0.466	−3.252	No	No	0.581	0.107	1.958	No	No	No
Quercetin	5280343	−2.925	77.20	−2.35	−1.098	−3.065	No	Yes	0.407	0.499	2.471	No	No	No
Xanthurenic acid	5699	−2.621	75.57	−2.35	−0.853	−3.313	No	No	0.553	1.039	2.805	Yes	No	No

BBB = blood brain barrier, CNSP = CNS permeability, IS = intestinal solubility, ORAT = oral rat acute toxicity, SP = skin permeability, TC = total clearance, WS = water solubility.

**Table 2:** Degree value of selected 18 compounds.

Compounds	Class	Degree	Compounds	Class	Degree
Catechin	Flavonoids	24	Gallic acid	Phenolic acids	5
Leucocyanidin	Flavonoids	24	Hydroquinone	Phenols	2
Alpha sitosterol	Sterol	14	Kaurenic acid	Diterpene	4
Apigenin	Flavonoid	35	Lupeol	Terpenoids	5
Beta amyrin	Terpenoids	14	Malic acid	Organic acids	2
Beta carotene	Carotenoid	6	Oleic acid	Fatty acids	7
Betulin	Terpenoids	5	Phloroglucinol	Phenols	2
Chlorogenic acid	Phenolic acids	6	Quercetin	Flavonoid	35
Ellagic acid	Phenolic acids	14	Xanthurenic acid	Quinoline carboxylic acid	2

**Table 3:** Binding energy and binding interaction mode of apigenin and quercetin.

Compounds	Compound-Target	Docking Score (kJ/mol)	Interaction			
			H-Bond Interactions			Arene-π
			Distance (ºA)	Score (%)	Amino Acid	
Apigenin	Apigenin-AKT1 Complex	−12.4640	3.15	15	LysA276	ArgB4
	Apigenin-EGFR Complex	−12.0665	3.02 2.41 2.93	41 40 42	AspB942 LysA757 LysA757	----
	Apigenin-VEGFA Complex	−12.8896	2.45 2.87	63 41	ThrA85 TyrA87	----
	Apigenin-Hsp90AB1 Complex	−14.7315	2.16 2.68	84 95	Ser52 Thr184	----
Quercetin	Quercetin-AKT1 Complex	−13.6098	2.87 3.12 2.9	91 10 35	Asp292 Thr291 Thr291	----
	Quercetin-EGFR Complex	−14.6961	1.43 2.94 2.59	24 39 85	AspA761 LysB949 LysB949	----
	Quercetin-VEGFA Complex	−15.1991	2.26 2.92 3.07 2.58	13 17 13 83	GlnB39 TyrA87 TyrA87 ThrA85	
	Quercetin-Hsp90AB1 Complex	−15.3669	3.01 1.91 2.75 2.89 1.93	22 14 18 30 75	Lys112 Asn51 Asn51 Ser52 Thr184	

## Data Availability

Not applicable.

## Supplementary Materials

The following supporting information can be downloaded at: https://www.mdpi.com/article/10.3390/life13020411/s1, Table S1, The names of active compound’s screening of AN; Table S2, compounds and target genes; Table S3, prospective asthma targets and Table S4, Overlapping genes between active ingredient targets with asthma targets.
